# YAP promotes the early development of temporomandibular joint bony ankylosis by regulating mesenchymal stem cell function

**DOI:** 10.1038/s41598-024-63613-8

**Published:** 2024-06-03

**Authors:** Tong-Mei Zhang, Mai-Ning Jiao, Kun Yang, Hua-Lun Wang, Chang-Song Zhang, Shi-Hua Wang, Guan-Meng Zhang, He-Jing Miao, Jun Shen, Ying-Bin Yan

**Affiliations:** 1 Tianjin Medical University Cancer Institute & Hospital, National Clinical Research Center for Cancer, West Huan-Hu Road, Ti Yuan Bei, Hexi District, Tianjin, 30060 China; 2 Tianjin’s Clinical Research Center for Cancer, West Huan-Hu Road, Ti Yuan Bei, Hexi District, Tianjin, 30060 China; 3 Key Laboratory of Cancer Prevention and Therapy, West Huan-Hu Road, Ti Yuan Bei, Hexi District, Tianjin, 30060 China; 4 Tianjin Medical University, 22 Qi-Xiang-Tai Road, Heping District, Tianjin, 300070 China; 5 Department of Oral and Maxillofacial Surgery, Weifang People’s Hospital, 151 GuangWen Street, KuiWen District, Weifang, 261100 ShanDong Province China; 6 Department of Oromaxillofacial-Head and Neck Surgery, China Three Gorges University Affiliated Renhe Hospital, 410 Yiling Ave, Hubei, 261100 China; 7 Department of Oral and Maxillofacial Surgery, Jining Stomatological Hospital, 22 Communist Youth League Road, Rencheng District, Jining, 261100 ShanDong Province China; 8 Department of Oromaxillofacial-Head and Neck Surgery, Tianjin Stomatological Hospital, School of Medicine, Nankai University, 75 Dagu Road, Heping District, Tianjin, 300041 China; 9 Tianjin Key Laboratory of Oral and Maxillofacial Function Reconstruction, 75 Dagu Road, Heping District, Tianjin, 300041 China; 10 Department of Operative Dentistry and Endodontics, Tianjin Stomatological Hospital, School of Medicine, Nankai University, 75 Dagu Road, Heping District, Tianjin, 300041 China; 11 Department of Stomatology Center, Shunde Hospital, Southern Medical University (The First People’s Hospital of Shunde), 1 Jiazi Road, Shunde District, Foshan, 528300 GuangDong Province China

**Keywords:** Temporomandibular joint ankylosis, Hippo/YAP, ShRNA, Trauma, Osteogenic, Sheep, Oral diseases, Trauma, Pathogenesis

## Abstract

To explore the role of YAP, a key effector of the Hippo pathway, in temporomandibular joint (TMJ) ankylosis. The temporal and spatial expression of YAP was detected via immunohistochemistry and multiplex immunohistochemistry on postoperative Days 1, 4, 7, 9, 11, 14 and 28 in a sheep model. Isolated mesenchymal stem cells (MSCs) from samples of the Day 14. The relative mRNA expression of YAP was examined before and after the osteogenic induction of MSCs. A YAP-silenced MSC model was constructed, and the effect of YAP knockdown on MSC function was examined. YAP is expressed in the nucleus of the key sites that determine the ankylosis formation, indicating that YAP is activated in a physiological state. The expression of YAP increased gradually over time. Moreover, the number of cells coexpressing of RUNX2 and YAP—with the osteogenic active zone labelled by RUNX2—tended to increase after Day 9. After the osteogenic induction of MSCs, the expression of YAP increased. After silencing YAP, the osteogenic, proliferative and migratory abilities of the MSCs were inhibited. YAP is involved in the early development of TMJ bony ankylosis. Inhibition of YAP using shRNA might be a promising way to prevent or treat TMJ ankylosis.

## Introduction

Temporomandibular joint (TMJ) ankylosis is caused by bony or fibrous adhesion of anatomic joint components resulting in progressive limitations in mouth opening^1,2^, and trauma is the main cause of this condition^3^. Because the TMJ is close to the skull base (i.e., the surrounding area is rich in blood vessels and nerves), treating TMJ ankylosis is challenging and technically demanding for surgeons.

To date, the exact molecular mechanisms underlying TMJ ankylosis remain unclear. TMJ ankylosis is an abnormal bone healing process, and an increasing number of studies have shown that the process of TMJ ankylosis formation is similar to that of hypertrophic nonunion^4,5^. Based on the results of several animal model experiments performed by our research group^6–8^, joint disc displacement and severe injury to the joint surface conjointly constitute the traumatic microenvironment of ankylosis formation. During ankylosis formation, mesenchymal stem cells (MSCs)^9–12^ especially MSCs from bony ankylosed calluses^13^ play a crucial role in TMJ ankylosis formation. Hence, inhibiting the osteogenic and chondrogenic differentiation of MSCs might be a promising strategy for preventing bony ankylosis in the TMJ.

Recent studies have shown that the Hippo signalling pathway is involved in bone development, bone regeneration and bone repair processes^14^. The Hippo signalling pathway is involved in the regulation of organ development, cell growth and development and tissue regeneration^15^, moreover, it can synergistically regulate the osteogenic differentiation of MSCs via other pathways^16,17^. In our previous study^18^, which is based on an analysis of temporal gene expression profiles, we found elevated Hippo signalling activity throughout the onset stage and early stage of traumatic TMJ bony ankylosis in a sheep model.

YES-associated protein (YAP) is understood to be a downstream effector of the Hippo pathway and is the principal regulator of organ growth during development. YAP localisation within the cell can reflect its physiological state^19^. When YAP is expressed in the nucleus, this indicates that YAP is activated and will interact with transcriptional enhanced associate domain (TEAD) factors or other transcription factors to regulate the downstream signalling cascade; when YAP fails to undergo nuclear translocation, it will be isolated in the cytoplasm, lose its role, and eventually degrade.

An increasing number of studies have shown that YAP, as the ‘switch’ of the Hippo signalling pathway, plays an important role in active osteogenic activity. YAP can facilitate MSC commitment to an osteoblastic lineage while suppressing adipogenesis^20^. Zhao et al.^21^ found that YAP is a necessary condition for cranial neural crest cells (NCCs) to produce craniofacial bone and cartilage in a normal and stable manner, and Hippo–YAP signalling promotes the osteogenesis of NCCs via interaction with the Wnt–β-catenin pathway. YAP inhibition during bone repair can lead to severe damage to BMP signalling pathways, thus inhibiting osteoblast differentiation^22^. Furthermore, YAP can regulate multiple steps of the chondrocyte differentiation process during bone repair via interactions with SOX6 and COL10A1^23^. Hippo–YAP signalling also has a regulatory effect on osteoclastogenesis and resorption activity. Zhao et al.^24^ reported that in macrophages derived from bone marrow, knockout of YAP prevents the formation and function of multinucleated osteoclasts and significantly decreases the expression of osteoclast marker genes.

Bone is a highly vascularized tissue with good vascular structure and blood circulation, guaranteeing normal and stable bone healing. YAP promotes sprouting angiogenesis by promoting the activity and function of blood vessel tip cells, and YAP has also been found to regulate metabolism and proliferation in vascular endothelial cells^25^. However, direct genetic evidence linking the Hippo–YAP signalling pathway to TMJ ankylosis development has not been reported.

To investigate whether YAP plays a role in the development of TMJ ankylosis, we examined YAP expression in histological sections from a sheep model of TMJ ankylosis at different time points. Furthermore, we isolated MSCs from newly generated ankylosed calluses, and quantified osteogenic gene expression in sheep MSCs in vitro after using short hairpin RNA (shRNA) technology to knock out YAP. We also evaluated the effects of shYAP on the proliferation and migration of MSCs.

## Materials and methods

### Animal model and tissue processing

Twenty-six 3-month-old male small-tailed sheep with body weights ranging from 25 to 27 kg were used in this study under a research protocol approved by the Ethics Committee of Tianjin Stomatological Hospital (approval code: Tjskq2013001). All experiments were performed in compliance with the Animal Management Regulations and Administrative Measures on Experimental Animals, and are reported in accordance with the ARRIVE guidelines. The animals were housed in a laboratory animal facility with adequate facility management services and specialised nursing care and husbandry practices, similar to our previous study^8^. The animals underwent unilateral TMJ surgery, involving the removal of two-thirds of the articular disc and severe damage to the articular fossa to induce bony ankylosis, following the protocol in our previous publication^8^. The anaesthesia, analgesia and euthanasia methods used were the same as those described in a previous study^26^.

Three sheep per time point were sacrificed for tissue specimen collection via euthanasia (120 mg/kg of pentobarbitone sodium administered via intravenous injection) on Days 1, 4, 7, 9, 11, 14 and 28 postsurgery, and five were sacrificed on Day 14 after TMJ surgery for subsequent isolation and culture of MSCs. The TMJ complexes were removed en bloc with a band saw, and newly formed tissue within the joint space was bluntly dissected from the surrounding soft tissue using periosteum separators.

### Histological evaluation and immunohistochemistry

After fixation with 10% formalin, the collected tissue was routinely dehydrated and embedded and then cut into 5 μm-thick sections using a microtome for subsequent staining experiments. Successive slices were taken to ensure that the haematoxylin and eosin (HE), immunohistochemistry (IHC) and multiplex immunohistochemistry (mIHC) staining were performed on the same area.

To observe the histological manifestations of the early stages of traumatic bony ankylosis of the TMJ, paraffin sections of tissues collected on Days 1, 4, 7, 9, 11, 14 and 28 post -operation were stained with HE (Sigma, USA).

IHC was used to detect YAP and Runt-related transcription factor 2 (RUNX2) expression positions in TMJ ankylosis formation. Antibodies against RUNX2 was used to label the osteogenically active regions. After routine dewaxing, hydration, heat repair and antibody blocking of the tissue sections, a nonblocking kit (ZSGBBIO, China) was used to prepare the sections for staining with an anti-YAP antibody (rabbit monoclonal, 1:1000, ProteinTech Group, USA) and an anti-RUNX2 antibody (mouse monoclonal, 1:1000, Abcam, USA), to which a matching secondary antibody was then added, followed by incubation at 37 °C for 30 min. Diaminobenzidine (DAB, 1:20, ZSGBBIO, China) was used for the final immunological colour development, and the nuclei were stained with haematoxylin (Sigma, USA). Image acquisition was performed using a microscope (Nikon, Japan).

### Multiplex immunohistochemistry (mIHC)

We used mIHC to further analyse the expression level of YAP in the early stages of traumatic bony ankylosis of the TMJ. A specialised tyramine signal amplification-immunohistochemistry (TSA-IHC) multitarget immunofluorescence staining kit (Bruno, China) was used for this experiment. Different primary antibodies were applied sequentially, followed by horseradish peroxidase-coupled secondary antibody incubation and tyramine signal amplification (TSA). After each TSA, the slides were processed for antigen elution. After labelling YAP and RUNX2, cell nuclei were stained with 4', 6-diamidino-2-phenylindole (DAPI) (Bruno, China). The images were acquired using a panoramic microscope camera system (Jinan Tangier Electronics Co., China), and the digital scanning and viewing software used was CaseViewer 2.4 (3DHISTECH, Hungary).

All collected tissues were initially viewed at × 100 for each section, in which functioning YAP was stained red, RUNX2 was stained green, and nuclei were stained blue. Subsequently, 20 × micrographs were acquired and three fields of view were obtained for each specimen. The percentage of cells exhibiting positive coexpression of RUNX2 + YAP (i.e., number of positive coexpression cells ÷ total number of cells) was calculated using the Halo (Indica Labs, USA) whole-slide image analysis platform. The fluorescence intensity measured as the integrated density (Int Den) and area were measured using ImageJ (National Institutes of Health, USA), and the mean grey value (mean) of each image was then calculated.

### Isolation and culture of MSCs

Primary cells were isolated using the tissue attachment method^27^. The specific isolation, incubation, culture and purification processes applied to the MSCs were the same as described in a previous study^13^. Third-passage cells were harvested for subsequent experimental procedures.

### Flow cytometry

The third-passage cells were digested with 0.25% trypsin (Solarbio, China) to obtain a single-cell suspension. The cells were then resuspended with phosphate buffered saline (PBS) solution supplemented with 1% foetal bovine serum (FBS) after centrifugation. For 30 min at 4 °C in the dark, 1 × 10^6^ cells were incubated with the corresponding commercial monoclonal antibodies (CD44, Immunostep, clone 25.32, 1:10; CD29, Biolegend, clone TS2/16, 1:20; CD31, AbD Serotec, clone CO.3E1D4, 1:10; CD45, AbD Serotec, clone 1.11.32, 1:10), and then measurements were made using a FACSCanto (BD Biosciences, USA) flow cytometer. Flow cytometric analyses were performed using FACSDiva (BD Biosciences, USA) and FlowJo software (TreeStar, Ashland, Oregon).

### Osteogenic differentiation evaluation

Third-passage cells were seeded at a density of 1 × 10^5^/ml in a six-well plate (Corning, USA). When the cells reached 60–70% confluence, the medium was removed, and the cells were washed with PBS three times. Osteogenic induction (OI) medium (the formula used for this osteogenic induction medium is described in an earlier study^13^) was added to the OI group, while normal control (NC) group was still added complete medium (α-minimal essential medium (α-MEM) plus 10% foetal bovine serum, 100 U/ml penicillin, 100 μg/ml streptomycin and 2.5 μg/ml amphotericin B).

The cells were stained with 1% Alizarin red (pH = 4.3, Solarbio, China) after undergoing OI for 7 days and, subsequently, 14 days. Mineralised nodules were then dissolved in 10% cetylpyridinium chloride (Solarbio, China) for semiquantitative analysis by examining the absorbance at 562 nm. Alkaline phosphatase (ALP) activity was tested using an ALP colour development kit (Beyotime, China) after the cells underwent OI for 7 days and, subsequently, 14 days. The mRNA expression levels of YAP, RUNX2, the Sp7 transcription factor (Osterix) and osteocalcin (OCN) were measured via real-time polymerase chain reaction (PCR) after induction for 7 and 14 days.

### Immunofluorescence

To detect the intracellular localisation of YAP during the OI of MSCs, third-passage cells were inoculated into confocal culture dishes (NEST, China) at a density of 1 × 10^3^ cells/ml, the OI medium was cultured for three days, and immunofluorescence staining was performed as described in a previous study^13^. The monoclonal antibody used was anti-mouse YAP1 (clone: 3A7A9, ProteinTech Group, 1:800) with 594-conjugated goat anti-mouse lgG. Fluorescently labelled cells were photographed using an inverted fluorescence microscope (Nikon, Japan).

### Reverse transcription and real‑time polymerase chain reaction (PCR)

Total cellular RNA was extracted using a universal RNA extraction kit (TaKaRa, Japan), and the RNA concentration was determined. cDNA was extracted via reverse transcription using a synthesis kit (Promega, USA), and quantitative real-time PCR was performed with FastStart Universal SYBR Green Master Mix (Roche, Switzerland, Cat. #04,913,850,001) using a LightCycler 480 II (Roche, Switzerland). Glyceraldehyde-3-phosphate dehydrogenase (GAPDH) was used as an internal reference. The reaction system and PCR cycling parameters were the same as those described in a previous study^28^. The sequences of the primers used for PCR amplification are listed in Table 1.

**Table 1:** Real-time PCR primer sequence.

Gene	Gene bank number	Primer sequences (5′–3′)	Product size (bp)
GAPDH	AF030943.1	GCAAGTTCCACGGCACAG	249 bp
		GGTTCACGCCCATCACAA	
YAP	NM_001267881.3	CCCTCGTTTTGCCATGAACC	207 bp
		CGGAGAGCTAATTCCTGCCG	
RUNX2	DY517479.1	TCGCCTCACAAACAACCA	102 bp
		AGGGACCTGCGGAGATTA	
OCN	DQ418490.1	AGATGCAAAGCCTGGTGATGC	211 bp
		CTCCTGGAAGCCGATGTGGT	
Osterix	NM_001102142.1	CAGCGGCGTGCAGTAAAT	240 bp
		CTGGGAACGAGTGGGAAAA	

### RNA interference

A YAP-silenced MSC model was constructed using shRNA plasmid transfection technology (RuiboBio, Guangzhou, China). The target sequences are presented in Table 2. Lipofectamine 3000 (Thermo Fisher Scientific, Waltham, USA) was used to transfect the MSCs, according to the manufacturer’s instructions. Third-passage cells were briefly inoculated into 35 mm dishes (Corning, USA) at a density of 1 × 10^5^ cells/ml after transient transfection with shYAP1, shYAP2, shYAP3 and shRNA plasmid empty vectors. The transfection efficiency was tested after 24 h of culture to determine the highest transfection efficiency of the shRNA plasmid. The MSCs were then reinoculated in 35 mm dishes following the previously described density and method, and divided into three groups: The blank group (the blank control group, in which the shRNA plasmid was not transfected), the shRNA-YAP-NC group (the negative control group, in which the shRNA plasmid was transfected with an empty vector), and the shRNA-YAP group (the silencing group, in which the shYAP plasmid was transfected with the highest efficiency). After one day of transfection, the stock solutions of the three groups were discarded, after the following experimental procedures: (1) The stock medium was replaced with OI medium to detect the effect of YAP silencing on the osteogenic ability of the MSCs; the procedures for Alizarin red staining, ALP activity detection and real-time PCR were the same as those described earlier. (2) Continue to use complete medium to detect the effects of YAP silencing on the proliferation and migration ability of MSCs.

**Table 2:** Designed YAP-shRNA sequence.

Gene		Target sequence
YAP	shYAP1	CACATAGATCAGACAACAA
	shYAP2	GCCATGAACCAGAGAATCA
	shYAP3	GGAGAAAGAAAGACTGCGA

### Cell proliferation assay

EdU assay, cell formation assays and cell counting kit-8 (CCK-8) assays were used to investigate whether YAP silencing affects the proliferative capacity of MSCs.

#### EdU assay

A BeyoClick EdU-488 Cell Proliferation Assay Kit (Beyotime, China) was used to determine the cell proliferative capacity. Third-passage MSCs were seeded in 24-well plates (Corning, USA) at a density of 2,000 cells /well. After transfection using the preceding method, the three groups of cells (Blank, shRNA-YAP-NC and shRNA-YAP) were stained following the protocol of the kit. Fluorescence detection was performed on each well at a 495 nm wavelength (× 10) using an inverted fluorescence microscope (Nikon, Japan). Quantitative analysis of EdU proliferation was performed using ImageJ; the percentage of EdU-positive = cells was calculated as (number of EdU-positive cells divided by the total number of cells) × 100%.

#### Cell colony formation assay

Third-passage cells were inoculated in 6-well plates (Corning, USA) at a density of 500 cells/well. After transfection using the method described earlier, the three groups of cells (Blank, shRNA-YAP-NC and shRNA-YAP) were fixed with 4% paraformaldehyde and stained with 5% Giemsa (Hydrogen, China) for 40 min on Day 7 of culture, and colonies containing > 50 cells were counted under the microscope (Nikon, Japan). To clearly observe cell proliferation under a microscope, the number of colonies was analysed using ImageJ.

#### Cell counting kit -8 (CCK-8) assay

Third-passage cells were seeded in four 96-well plates (Corning, USA) at a density of 2,000 cells/well. After transfection following the method described earlier, cell proliferation tests were performed on the three groups of cells (Blank, shRNA-YAP-NC and shRNA-YAP) using a CCK-8 kit (Beyotime, China) as described in a previous study^13^, and the detection time points were 1 day, 3 days, 5 days and 7 days.

### Cell migration and invasion assays

Wound healing and Transwell migration assays were used to investigate whether YAP silencing affects the migratory capacity of MSCs.

#### Wound healing assay

Third-passage MSCs were inoculated in 6-well plates (Corning, USA) at a density of 1 × 10^5^ cells /well. When the cells reached to 90% confluence after transfection, a straight line was drawn in the cell layer with a sterile 200 μl pipet tip. Images were acquired under a microscope (Nikon, Japan) at 0 h, 6 h, 24 h and 48 h. The distance migrated by the cells was analysed using ImageJ. The relative wound closure rate was calculated as the ratio of the wound distance at 6 h, 24 h and 48 h to the wound distance at 0 h.

#### Transwell migration assay

A 24‑well plate with Transwell chambers (Beyotime, China) with an 8.0 µm pore size was used for this experiment. Serum‑free α-MEM was added to the upper chambers, and a medium containing 20% FBS was added to the lower chamber. After transfection, the cells of the three groups (Blank, shRNA-YAP-NC, shRNA-YAP) were inoculated in the upper chamber at a cell density of 3 × 10^4^/well. After 24 h of incubation at 37 °C, the migratory cells on the membrane were fixed in 4% paraformaldehyde and stained with 0.1% crystal violet (Beyotime, China) for 30 min. The membrane was air-dried and then photographed under a light microscope (Nikon, Japan) at 10 × magnification. The number of migrated cells was quantified using ImageJ.

### Statistical analysis

The results of the experiments were analysed using unpaired t tests between two groups, while comparisons among three or more groups were performed using one-way ANOVA combined with Bonferroni’s multiple comparisons test (GraphPad Prism 9.0, USA); *P* < 0.05 was considered to indicate statistical significance. The D’Agostino-Pearson test was used to assess normality. Lines and error bars in all figures denote of the mean and standard error mean (SEM), respectively.

## Results

### YAP expression and activation in a sheep model of surgery-induced bony ankylosis of the TMJ

**Figure 1:**
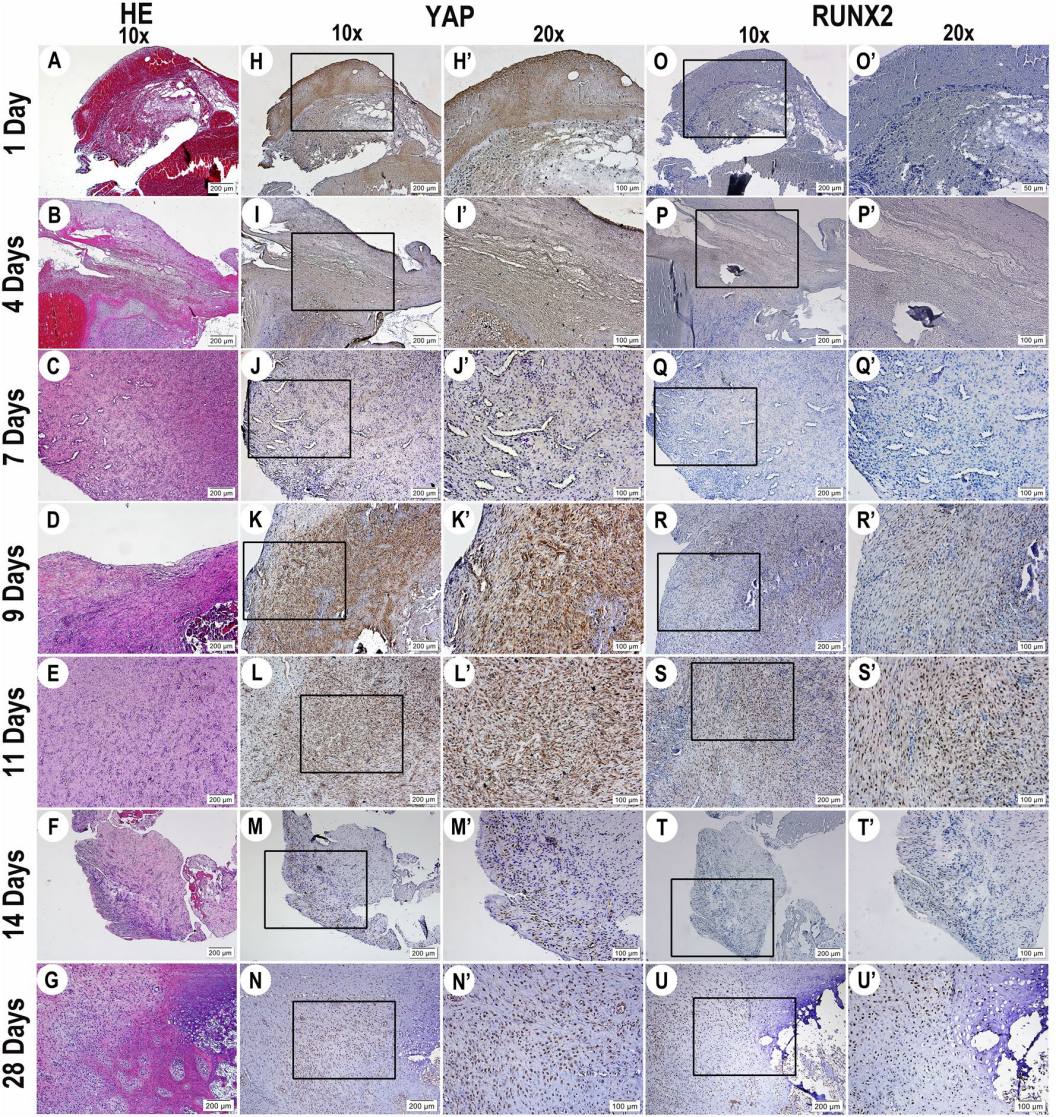
YAP expression and activation during early-stage TMJ bony ankylosis in a sheep model. (**A**–**G**) HE staining highlights the newly generated ankylosed callus on Days 1, 4, 7, 9, 11, 14 and 28. (**H**–**N**, **H’**–**N’**) IHC reveals the expression and distribution of YAP in the newly generated ankylosed callus on Days 1, 4, 7, 9, 11, 14 and 28. (**O**–**U**, **O’**–**U’**) IHC reveals the expression and distribution of RUNX2 in the newly generated ankylosed callus on Days 1, 4, 7, 9, 11, 14 and 28. *TMJ* temporomandibular joint, *HE* haematoxylin and eosin, *IHC* immunohistochemistry. (**A**–**U**) magnification =  × 10, scale bar = 200 μm. (**H’**–**U’**) magnification =  × 20, scale bar = 100 μm.

According to the histological results (Fig. 1) of the tests performed on traumatic TMJ ankylosis in the first month in the sheep model, we found that YAP was expressed during the ankylosis process and that it was activated, indicating that YAP participated in the early development of bony ankylosis in the TMJ.

On the first day after surgery, a haematoma accompanied by an acute inflammatory response was observed in the TMJ area, with infiltration of many inflammatory cells, the main functions of these cells were phagocytosis and removal of necrotic material (Fig. 1A). YAP was also located in the nucleus at the site of acute inflammation (Fig. 1H,H’). On the fourth day, the type of inflammatory cells changed, the inflammatory response decreased (Fig. 1B), and YAP was located in the nucleus at the site of inflammatory exudation (‘Fig. 1I,I’). On Day 7, the haematoma was continuously organised and replaced by fibrovascular tissue to form granulation tissue (Fig. 1C). Interestingly, YAP was located in the nucleus at the granulation tissue site at this time (Fig. 1J,J’). The osteogenic marker RUNX2 was almost completely absent from Day 1 to Day 7 postsurgery (Fig. 1O,O’,P, P’,Q,Q’).

Gradually deposited collagen fibres appeared on Day 9 and Day 11 (Fig. 1D,E), and YAP was located in the nucleus at the site of collagen fibre formation (Fig. 1K,K’,L,L’). Starting on Day 9 (Fig. 1R,R’,S,S’), RUNX2 was positively expressed in some regions, and its expression levels gradually increased over time.

Chondrocytes appeared on Day 14 (Fig. 1F) and YAP (Fig. 1M,M’) and RUNX2 (Fig. 1T,T’) showed significant coexpression in this region, indicating the commencement of cartilaginous internalisation of bone. On Day 28, cartilage had formed locally (Fig. 1G), and co-expression of YAP (Fig. 1N,N’) and RUNX2 (Fig. 1U,U’) in the nuclei of the hypertrophic chondrocytes was more evident.

### YAP levels increased gradually during TMJ ankylosis development, with expression in the osteogenic region beginning on Day 9

**Figure 2:**
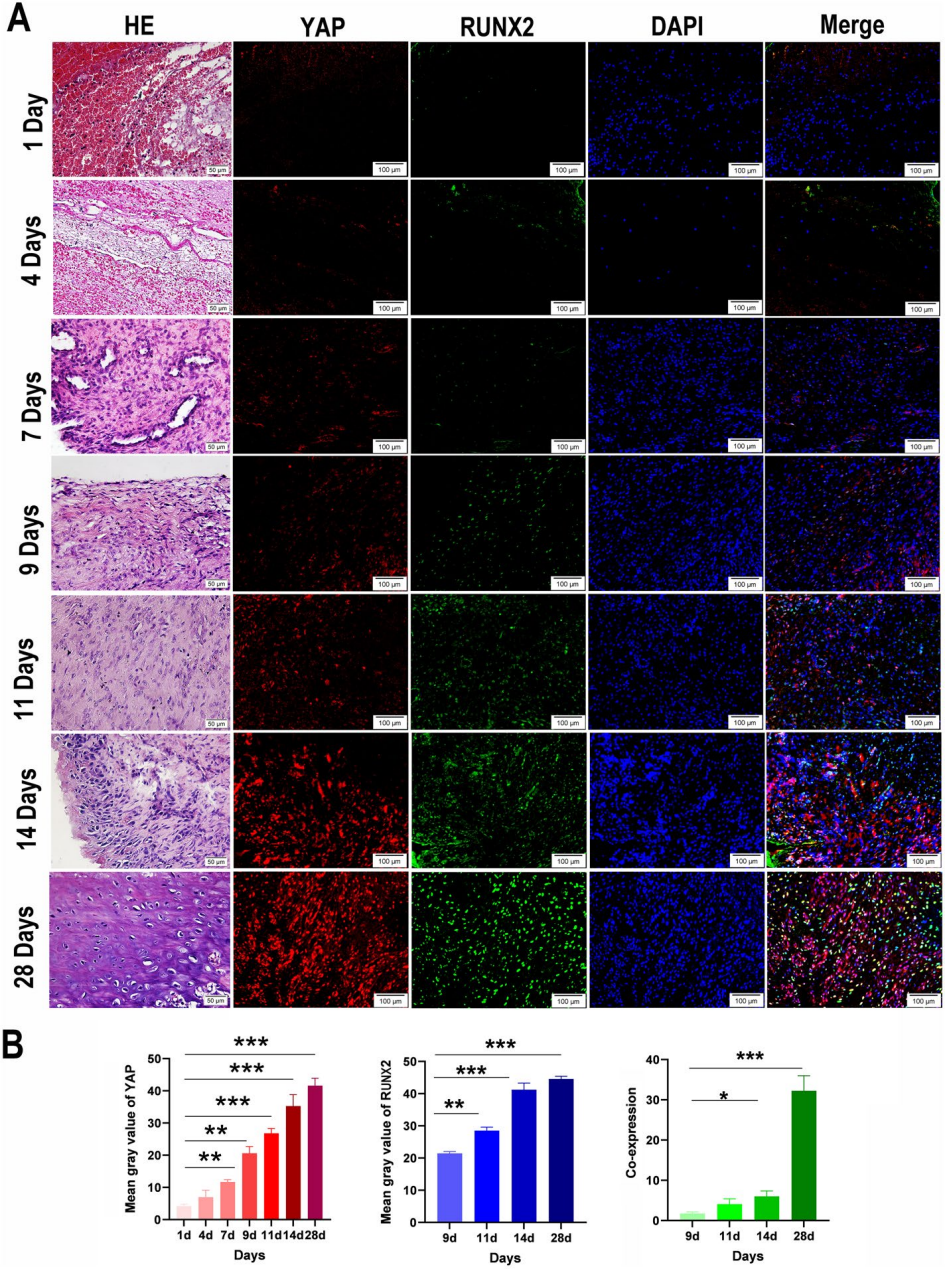
The level of YAP gradually increases with increasing YAP expression beginning in the bone formation area on Day 9 during TMJ ankylosis development in a sheep model. (**A**) Representative mIHC images of the newly generated ankylosed callus and HE images at the corresponding timepoints on Days 1, 4, 7, 9, 11, 14 and 28 (red: YAP; green: RUNX2; blue: DAPI; cells exhibiting positive coexpression of RUNX2 and YAP are shown in the merged image; HE images: scale bar = 50 μm; mIHC images: scale bar = 100 μm). (**B**) Based on the mIHC image, the immunofluorescence intensity of YAP shows a gradually increasing trend throughout the process, and the immunofluorescence intensity of RUNX2 and the copositive RUNX2 and YAP cells show an increasing trend from Day 9 (three images per three biological replicates for each timepoint, *P < 0.05, **P < 0.01, ***P < 0.001). *TMJ* temporomandibular joint, *HE* haematoxylin and eosin, *mIHC* multiplex immunohistochemistry.

We validated the immunohistochemistry results by conducting mIHC staining of traumatic bony ankylosis in the TMJ during the first month in the sheep model (Fig. 2A). Multiplex staining of YAP, RUNX2 and DAPI was performed on a single histological section of the collected specimen. The immunofluorescence intensity of YAP exhibited a gradually increasing trend throughout the process, and peaked on Day 28 (Fig. 2B). Compared with those on Day 1, the immunofluorescence intensities on Day 7 (*P* < 0.01), Day 9 (*P* < 0.01), Day 11 (*P* < 0.001), Day 14 (*P* < 0.001) and Day 28 (*P* < 0.001) were statistically significantly different.

The immunofluorescence intensity of RUNX2 exhibited an increasing trend beginning on Day 9 (Fig. 2B). Compared with those on Day 9, the immunofluorescence intensities on Day 11 (*P* < 0.01), Day 14 (*P* < 0.001) and Day 28 (*P* < 0.001) were significantly different.

In addition, strong interactions were observed between YAP and RUNX2. Compared with those on Day 9, the number of cells exhibiting positive coexpression of RUNX2 and YAP increased significantly on Day 14 (*P* < 0.05) and Day 28 (Fig. 2B), indicating that YAP expression began in the bone formation area on Day 9.

### Cell isolation and morphological characterisation

**Figure 3:**
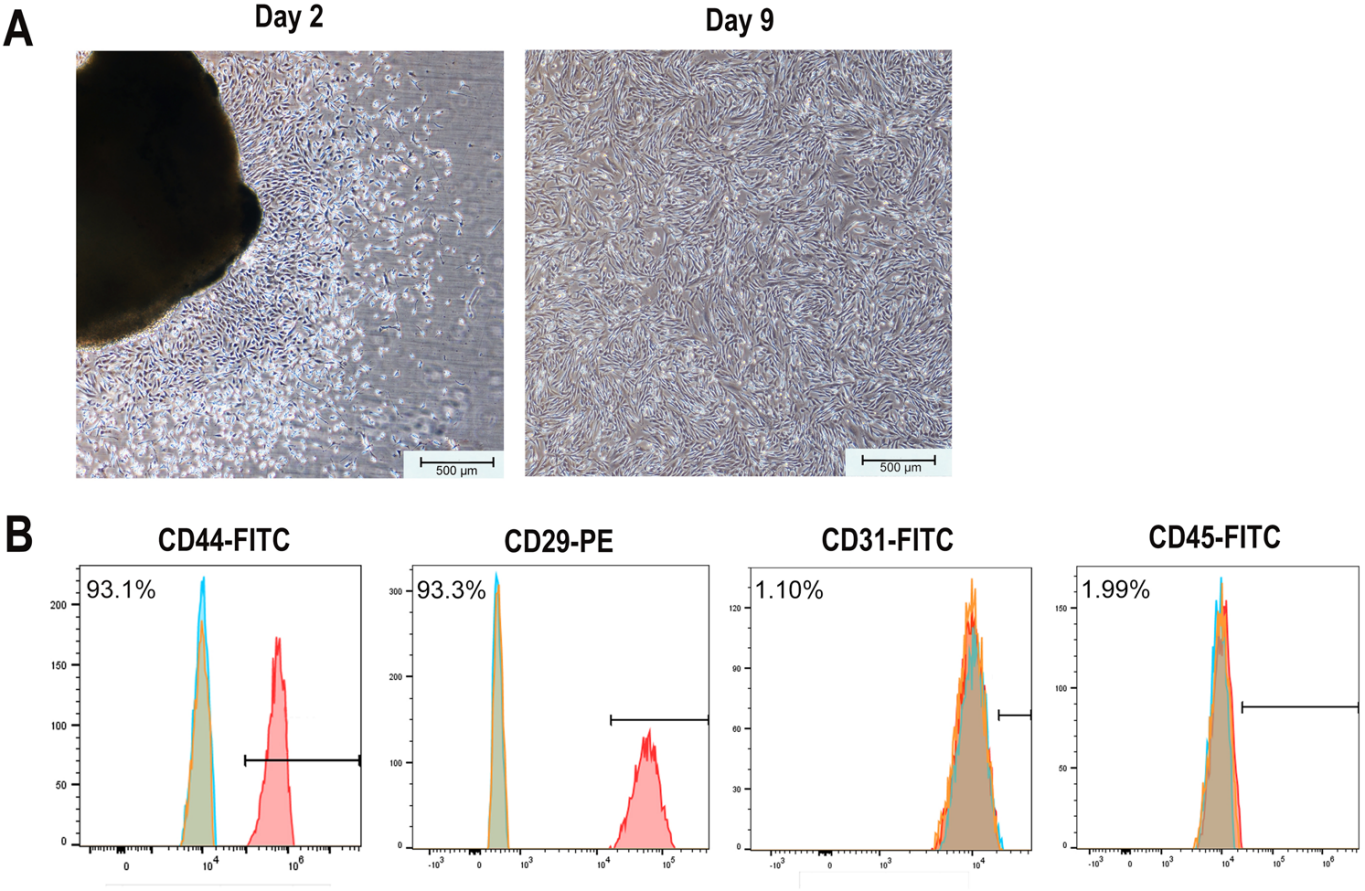
Cell culture, morphology and immunophenotyping of MSCs. (**A**) MSCs exhibit spindle- or fibroblast-like characteristics on Days 2 and 9 (scale bar = 500 μm). (**B**) Immunophenotypic characterisation of MSCs by flow cytometry. The third-passage cells were incubated with monoclonal antibodies against the cell-surface marker antigens CD44, CD29, CD31, and CD45, followed by fluorescein-conjugated secondary antibodies. *MSCs* mesenchymal stem cells.

Characteristic spindle-shaped or fibroblast-like adherent cell cultures were established from an ankylosed callus (Fig. 3A). The cells completely adhered to plastic and stretched after 1–2 days, reaching approximately 90% confluency after 9 days (Fig. 3A).

### Immunophenotypic characterisation of MSCs

The MSCs exhibited high expression levels of the MSCs markers CD44 (93.1%) and CD29 (93.3%). Furthermore, the MSCs were negative for CD31 (1.10%) and CD45 (1.99%) expression, indicating that there was no contamination with cells of haematopoietic origin (Fig. 3B).

### Osteogenic differentiation ability of MSCs

**Figure 4:**
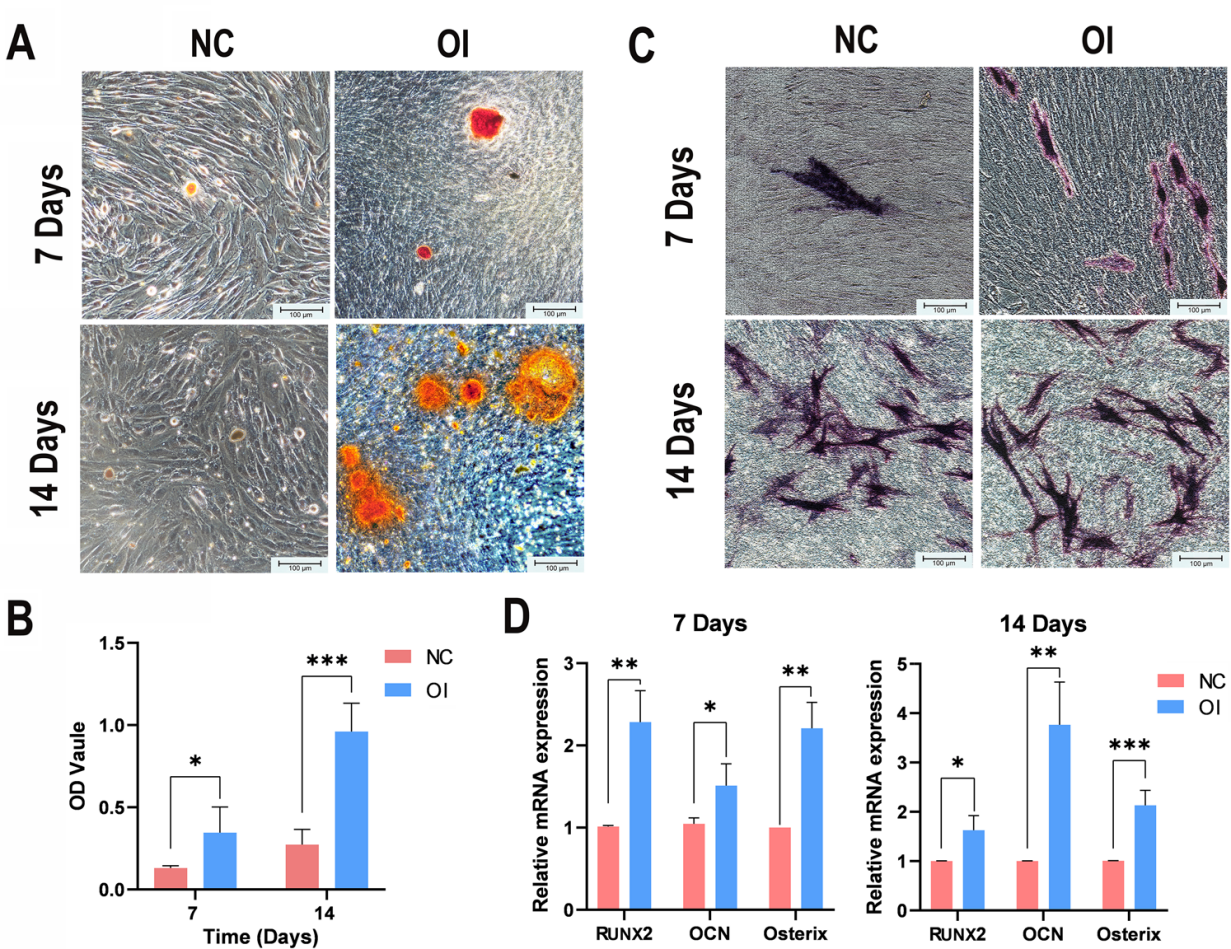
Osteogenic differentiation ability of MSCs. (**A**) The area of the mineralised calcium nodules detected using alizarin red staining at 7 days and 14 days of OI was larger in the OI group than in the NC group (scale bar = 100 μm). (**B**) Bar graph presenting the Alizarin red semi-quantitative analysis (n = 3, *P < 0.05, ***P < 0.001). (**C**) After 7 days and 14 days of OI, the ALP activity of MSCs detected via ALP staining in the OI group was greater than that detected in the NC group (scale bar = 100 μm). (**D**) mRNA expression of the osteogenesis-related genes RUNX2, OCN and Osterix in MSCs for the OI and NC groups on Day 7 and Day 14 (n = 3, *P < 0.05, **P < 0.01, ***P < 0.001). *OI* osteogenic induction, *NC* negative control, *MSCs* mesenchymal stem cells, *ALP* alkaline phosphatase.

The osteogenic ability of the MSCs was detected using alizarin red staining. After 7 days and subsequently 14 days of OI, the area of the calcium nodules in the OI group was significantly larger than that in the NC group (Fig. 4A). In addition, a semiquantitative analysis based on the Alizarin red staining (Fig. 4B) revealed that the difference was statistically significant (7 days: *P* < 0.05, 14 days: *P* < 0.001).

The osteogenic differentiation of the MSCs was also evaluated via ALP activity on Day 7 and Day 14 (Fig. 4C). Similarly, ALP staining was more obvious in the OI group than in the NC group.

The expression levels of three osteogenic lineage-specific genes (RUNX2, OCN and Osterix) were detected using real-time PCR (Fig. 4D). After seven days of OI, the OI group showed significantly greater RUNX2 (2.25-fold, *P* < 0.01), OCN (1.42-fold, *P* < 0.01) and Osterix (2.21-fold, *P* < 0.01) expression than the NC group (Fig. 4D). After 14 days of OI, the expression of RUNX2 (1.63-fold, *P* < 0.05), OCN (3.77-fold, *P* < 0.01) and Osterix (2.13-fold, *P* < 0.001) in the OI group was significantly greater than that in the NC group.

### Activation and upregulation of YAP during MSC osteogenic differentiation

**Figure 5:**
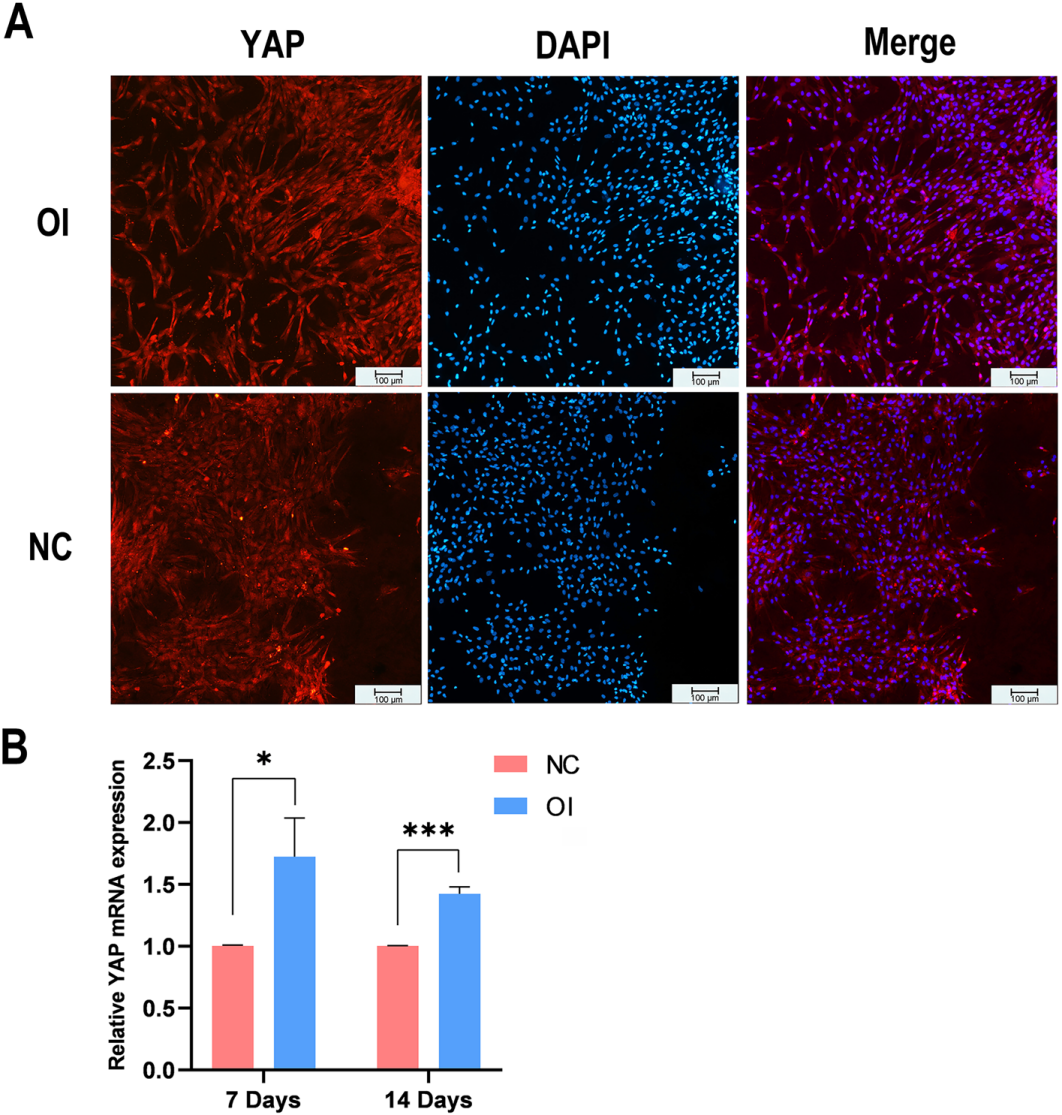
YAP activation and upregulation during MSC osteogenic differentiation. (**A**) Intracellular localisation of YAP detected via immunofluorescence in the NC and OI groups. In the OI group, YAP is more strongly expressed in the nucleus; the nuclei were stained blue by DAPI (scale bar = 100 μm). (**B**) YAP mRNA expression in MSCs in the OI and NC groups on Day 7 and Day 14. OI: osteogenic induction, *NC* negative control, *MSCs* mesenchymal stem cells.

The intracellular localisation of YAP was detected via immunofluorescence to determine whether YAP was activated during the osteogenic differentiation of MSCs (Fig. 5A). After three days of OI, YAP was expressed in the cytoplasm and nucleus of MSCs in both the NC group and the OI group. However, YAP was more strongly expressed in the cytoplasm in the NC group than in the OI group, and it was more strongly expressed in the nucleus in the OI group than in the NC group. This phenomenon indicates that OI fluid expedited the entry of YAP into the nucleus, i.e., YAP was activated during the osteogenic differentiation of MSCs.

In addition, YAP mRNA levels on Day 7 (1.72-fold, *P* < 0.05) and Day 14 (1.42-fold, *P* < 0.001) were significantly greater in the OI group than in the NC group (Fig. 5B), which indicates that YAP activation is a characteristic event during TMJ ankylosis development.

### YAP blockade inhibits the osteogenic ability of MSCs

**Figure 6:**
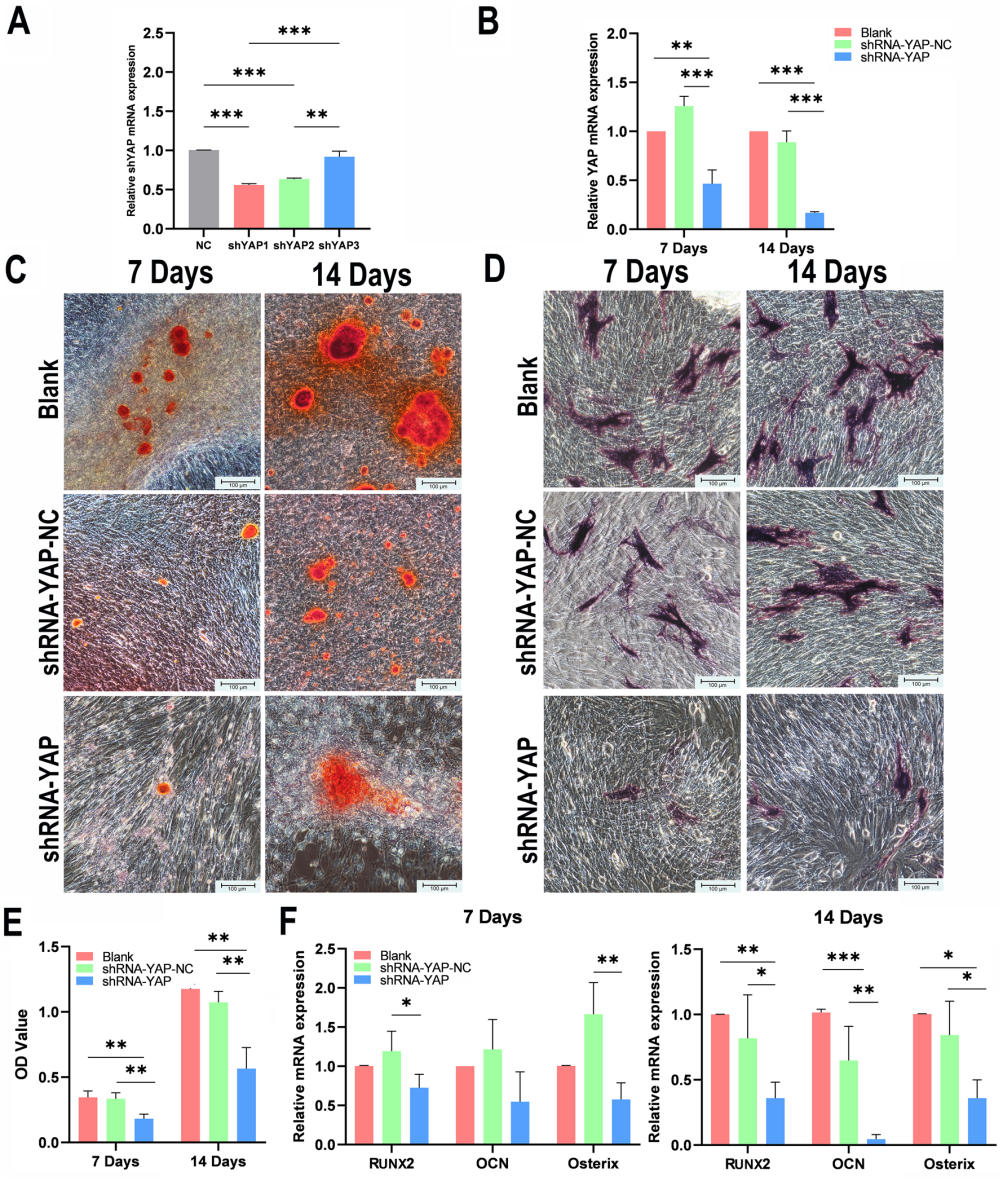
YAP blockade inhibits the osteogenic ability of MSCs. (**A**) Efficiency of three sequences of YAP shRNA obtained via real-time PCR. (**B**) YAP expression after OI of MSCs in the presence of shRNA plasmid empty vector (shRNA-YAP-NC), or YAP shRNA (shRNA-YAP) or in the absence of shRNA (Blank) for 7 days; subsequently, 14 days were assessed using real-time PCR (n = 3, **P < 0.01, ***P < 0.001). (**C**) After blocking YAP with shYAP, the area of the calcium nodules detected via Alizarin red staining decreased (scale bar = 100 μm). (**D**) After blocking YAP with shYAP, the ALP activity detected via ALP staining decreased (scale bar = 100 μm). (**E**) Alizarin red semiquantitative analysis of the shRNA-YAP-NC, shRNA-YAP and Blank groups (n = 3, **P < 0.01). (**F**) mRNA expression of the osteogenesis-related genes RUNX2, OCN and Osterix in the shRNA-YAP-NC, shRNA-YAP and Blank groups on Day 7 and Day 14 (n = 3, *P < 0.05, **P < 0.01, ***P < 0.001). *MSCs* mesenchymal stem cells, *ALP* alkaline phosphatase.

To further analyse the effect of YAP on the OI of MSCs, MSCs were transfected with YAP shRNA. We designed three YAP shRNAs, and for the next set of experiments (Fig. 6A), we selected the most efficient YAP shRNA (shYAP1) based on real-time PCR analysis.

To verify that the effect of YAP silencing was always present throughout the OI process, real-time PCR was used to detect YAP mRNA expression levels during the induction process (Fig. 6B). After the MSCs were transfected with shYAP and subjected to OI for seven days, the expression levels of YAP in the shRNA-YAP group were significantly lower than those in the Blank group (0.47-fold, *P* < 0.01) and the shRNA-YAP-NC group (0.37-fold, *P* < 0.001). The YAP expression levels in the Blank group and the shRNA-YAP-NC group were similar, with no statistically significant difference. Similarly, after 14 days of induction, the shRNA-YAP group exhibited a lower level of YAP expression than the Blank group (0.17-fold, *P* < 0.001) and the shRNA-YAP-NC group (0.19-fold, *P* < 0.001), and there was no significant difference in YAP expression between the Blank group and the shRNA-YAP-NC group.

After 7 days and subsequently 14 days of OI, the area of the calcium nodules in the shRNA-YAP group was significantly smaller than that in the Blank group and the shRNA-YAP-NC group (Fig. 6C). In addition, a semiquantitative analysis based on mineralised nodule staining (Fig. 6E) revealed that the differences were statistically significant (7 days: shRNA-YAP vs. Blank, *P* < 0.01; shRNA-YAP vs. shRNA-YAP-NC, *P* < 0.01. 14 days: shRNA-YAP vs. Blank, *P* < 0.01; shRNA-YAP vs. shRNA-YAP-NC, *P* < 0.01). Furthermore, no statistically significant difference was found between the calcium nodules area in the Blank group and that in the shRNA-YAP-NC group.

ALP staining revealed trends consistent with those of Alizarin red staining, and the area stained for osteogenic-induced MSCs (after 7 days and 14 days) in the shRNA-YAP group was smaller than that in the Blank group and the shRNA-YAP-NC group (Fig. 6D).

Furthermore, shYAP partially suppressed the increase in RUNX2, OCN and Osterix mRNA expression induced by OI (Fig. 6F). After seven days of OI, we found that the expression levels of RUNX2 (0.61-fold, *P* < 0.05) and Osterix (0.35-fold, *P* < 0.01) were significantly lower than those in the shRNA-YAP-NC group, and OCN expression in the shRNA-YAP groups tended to be lower than that in the Blank group and the shRNA-YAP-NC group, however, the differences were not statistically significant. Furthermore, after 14 days of induction, compared with those in the Blank group, the levels of RUNX2, OCN and Osterix in the shRNA-YAP group were decreased by 0.36-fold (*P* < 0.01), 0.04-fold (*P* < 0.001), and 0.36-fold (*P* < 0.05), respectively, and compared with those in the shRNA-YAP-NC group, the levels of RUNX2, OCN and Osterix in the shRNA-YAP group was decreased by 0.44-fold (*P* < 0.05), 0.70-fold (*P* < 0.01) and 0.43-fold (*P* < 0.05), respectively.

### YAP knockdown decreases MSC proliferation

**Figure 7:**
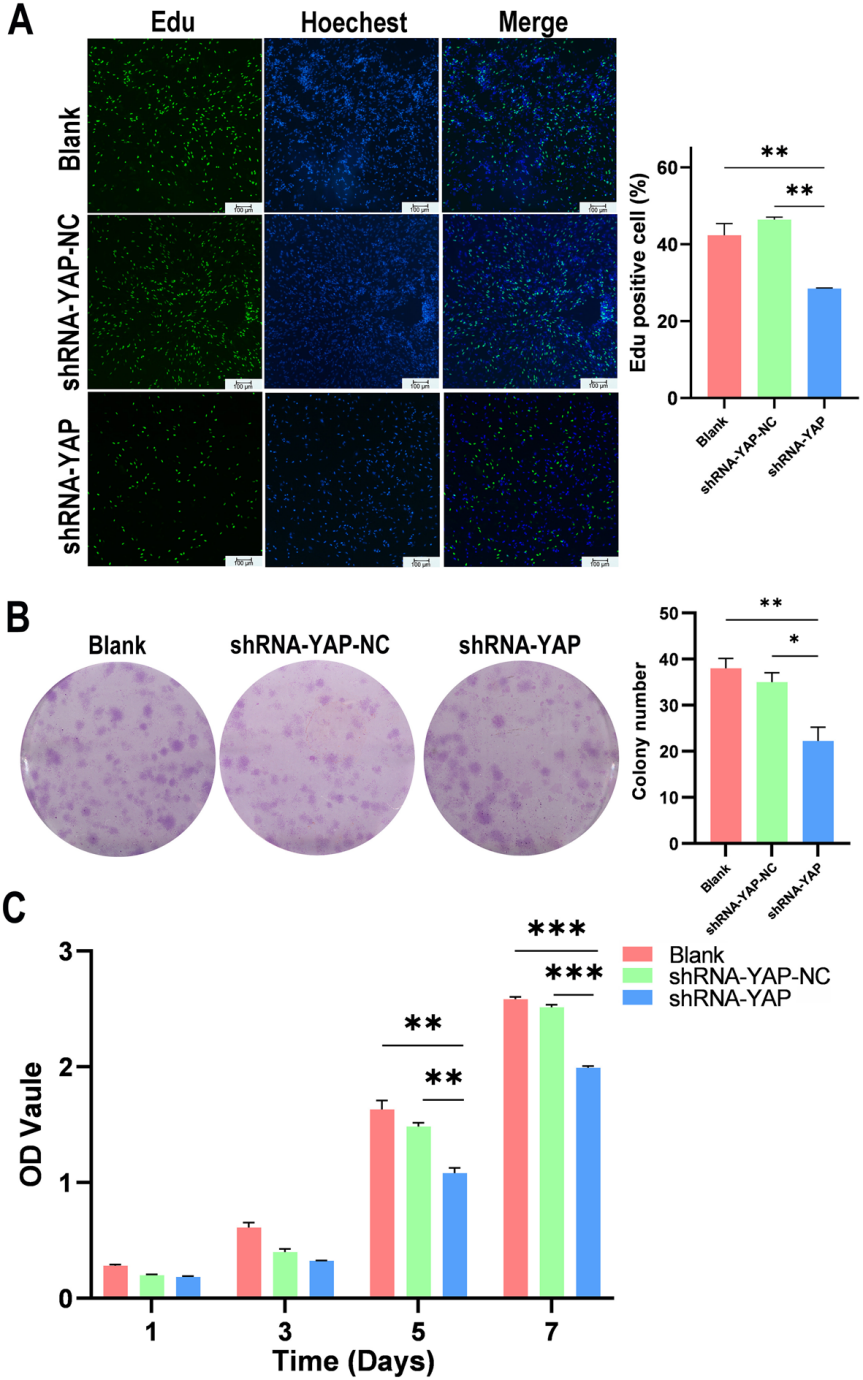
YAP knockdown reduced the proliferation ability of MSCs. (**A**) The EdU assay shows that the proliferation rate of MSCs with silenced YAP was significantly reduced (scale bar = 100 μm, n = 3, **P < 0.01). (**B**) The clonogenic potential of MSCs in which YAP was silenced is significantly lower, as determined by violet crystal staining and cell colony number (scale bar = 100 μm, n = 5, **P < 0.01). (**C**) MSCs with silenced YAP show decreased proliferation from Days 5 to 7 according to the CCK-8 assay (n = 5, **P < 0.01, ***P < 0.001). *MSCs* mesenchymal stem cells.

The percentage of EdU-positive cells (Fig. 7A) in the shRNA-YAP group was lower than that in the Blank group and shRNA-YAP-NC group (*P*_Blank_ < 0.01, *P*_shRNA-YAP-NC_ < 0.01). After seven days, single-cell colonies were observed via crystal violet staining (Fig. 7B) There were significantly fewer cell colonies in the shRNA-YAP group than in the Blank group (*P* < 0.01) and the shRNA-YAP-NC group (*P* < 0.05). Using the CCK-8 method, a cell proliferation curve was drawn based on the absorbance at each checkpoint (Fig. 7C). The curves show a similar proliferative potential for MSCs in the three groups from Day 1 to Day 3; however, there was a significantly lower viability in the shRNA-YAP group than in the Blank and shRNA-YAP-NC groups from Day 5 (*P*_Blank_ < 0.01, *P*_shRNA-YAP-NC_ < 0.01) to Day 7 (*P*_Blank_ < 0.001, *P*_shRNA-YAP-NC_ < 0.001).

### Silencing YAP suppresses MSC migration

**Figure 8:**
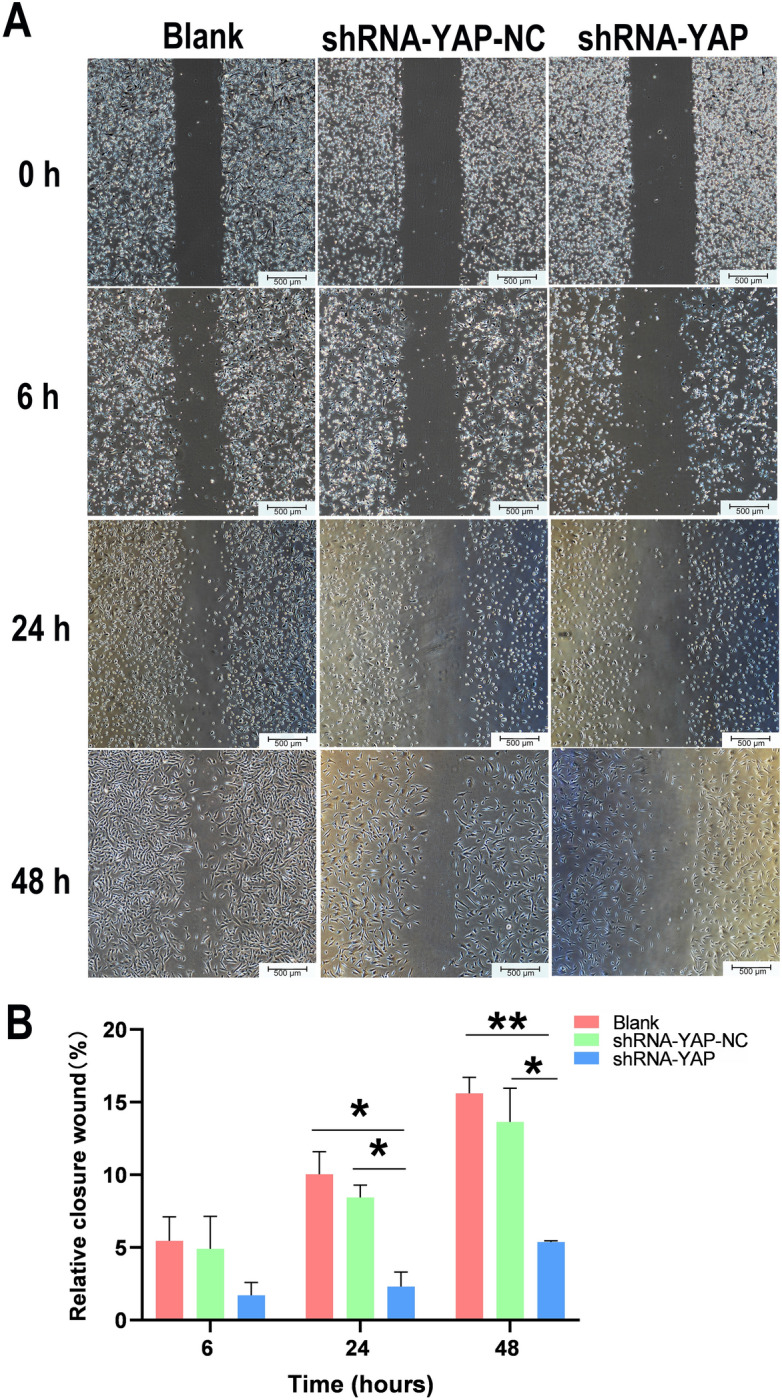
A wound healing assay shows that the migration ability of YAP-silenced MSCs is significantly reduced. (**A**) Wound healing assay under a microscope (scale bar = 500 μm). (**B**) The graphical data represent the percentage of the migration area determined via the wound healing assay (n = 3, *P < 0.05, **P < 0.01). *MSCs* mesenchymal stem cells.

**Figure 9:**
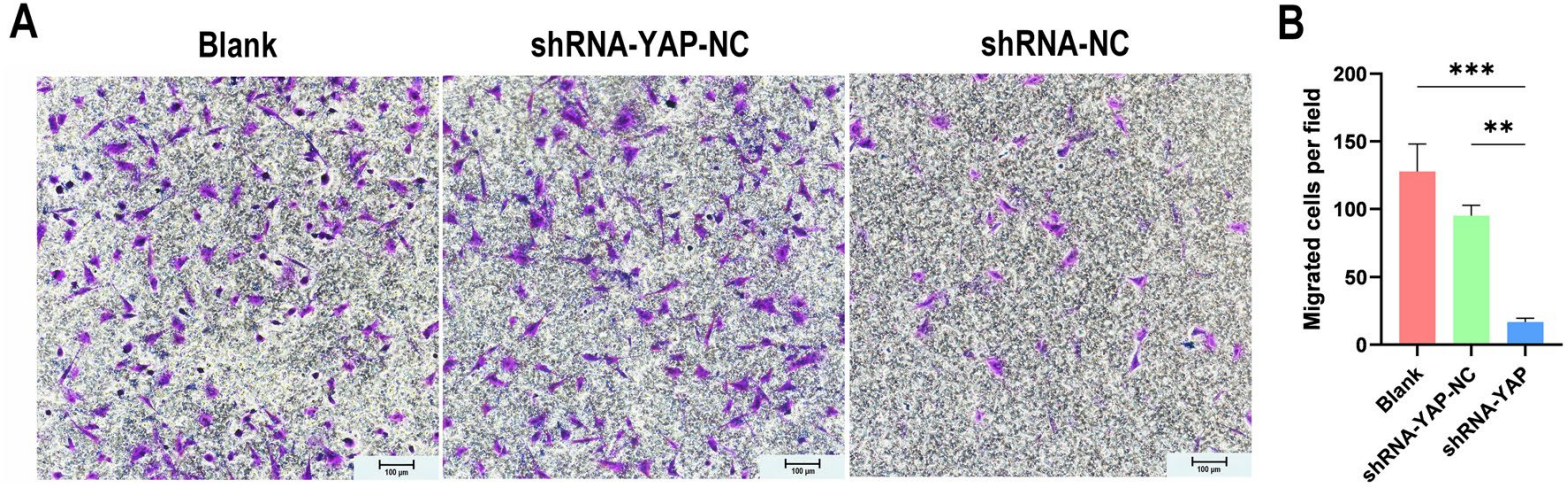
Transwell migration assays show that the migration ability of YAP-silenced MSCs is significantly reduced. (**A**) Transwell migration assay under a microscope (scale bar = 100 μm). (**B**) The graphical data represent the number of migrated cells per field (n = 4, **P < 0.01, ***P < 0.001). *MSCs* mesenchymal stem cells.

Based on the wound healing assay (Fig. 8), we found that the wound closure rate in the shRNA-YAP group was significantly lower than that in the shRNA-YAP-NC and Blank groups from 24 h (*P*_Blank_ < 0.05, *P*_shRNA-YAP-NC_ < 0.05) to 48 h (*P*_Blank_ < 0.01, *P*_shRNA-YAP-NC_ < 0.05). Moreover, the results of the Transwell migration assay (Fig. 9) showed similar trends with statistically significant differences (*P*_Blank_ < 0.001, *P*_shRNA-YAP-NC_ < 0.01), indicating that transfection of shYAP can lower the migration potential of MSCs.

## Discussion

TMJ ankylosis is a severely deforming, disabling condition; however, the pathological mechanisms involved in the progression of TMJ ankylosis have not been clearly elucidated. In this study, we first revealrevealed that Hippo–YAP signals playsignalling plays a crucial role in TMJ ankylosis formationdevelopment. We observed that precise regulation of YAP is critical for the osteogenic differentiation of MSCs in the early phases of TMJ bony ankylosis for osteogenic differentiation of MSCs, and itYAP can also expedite the proliferation and migration of MSCs.

In this study, we first described the distribution of YAP during ankylosis development in an animal model. Based on the HE manifestations of the model, we found that the development of TMJ ankylosis progressed through several stages, including blood clot formation (Day 1), inflammatory infiltration (Day 4), granulation tissue formation (Day 7), blood vessel and fibrous collagen tissue formation (Days 9–11), and new bone formation (Days 14–28).

The Hippo–YAP signalling pathway has been shown to be involved in various physiological activities involved in posttraumatic repair. In the early stage of trauma, YAP is involved in the survival, migration and proliferation of endothelial cells while simultaneously reducing endothelial inflammation^29^. As the haematoma and acute inflammation resolve, granulation begins. At this stage, YAP regulates vessel sprouting, vascular barrier formation and vascular remodelling^30^. As time progresses, the number of fibroblasts increases and collagen secretion begins^31,32^. In this phase, YAP is involved in the deposition of collagen fibres. With the continuous deposition of fibres, osteoblast-like cells appear and the initial stage of new bone formation commences. During this stage, YAP activity is ongoing, and YAP is involved in various signal transduction pathways during osteogenesis^14^. Bone morphogenetic protein 2 (BMP2) induces the phosphorylation of SMAD1/5/8 and then integrates with SMAD4 in the nucleus. Subsequently, YAP interacts with the SMAD complex to exert its transcriptional activation effect and induce the osteogenic differentiation of osteoprogenitor cells via RUNX2^33^. Our results showed that YAP is expressed in key regions at different time points during the development of traumatic TMJ ankylosis: YAP was found in the nuclei at the inflammation site from Day 1 to Day 4, in the nuclei at the granulation tissue site on Day 7, in the nuclei at the collagen fibre formation site from Day 9 to Day 11, and in the nuclei of the chondrocytes on Day 14 to Day 28 after surgery. It is likely that YAP is involved in every pathological stage of traumatic TMJ ankylosis. The mIHC results also verified that YAP was expressed and located in the nucleus at each time point.

Our results showed that YAP expression in early-phase ankylosis possibly also initiates the TMJ bone mass increase. RUNX2, a transcription factor belonging to the RUNX family, is expressed in MSCs, osteoblast lineage cells and chondrocytes^34^, and its expression is upregulated in preosteoblasts^35^; hence, it is often used as a marker of osteogenesis. In this study, mIHC staining was performed on YAP and RUNX2, and the RUNX2 staining area was used as an *osteogenic marker* to observe the distribution of YAP in the RUNX2-labelled area. The immunofluorescence intensity and number of cells positive for YAP and RUNX2 were quantitatively analysed. Thus, the expression trends and levels of Hippo–YAP signalling in traumatic TMJ bony ankylosis calluses were determined. Positive expression of RUNX2 was not observed until Day 9. Compared with those on Day 9, the number of cells exhibiting positive coexpression of RUNX2 and YAP was significantly different on Day 14 and Day 28: these genes were significantly increased. This indicates that YAP began to maintain the osteogenic reserve on Day 9 and was actively involved in osteogenesis on Day 14 and Day 28.

We found that Hippo–YAP signalling initiates bone mass formation by promoting the osteogenic differentiation of MSCs in TMJ ankylosis. OI promoted the nuclear translocation of YAP in MSCs and increased YAP mRNA expression levels, while silencing Hippo–YAP signalling inhibited the expression of the osteoblast-related markers OCN, RUNX2 and Osterix; the formation of mineralised nodules; and ALP activity in MSCs. As reported in our previous study^4,7^, after trauma, the presence of cyclic shear stress in the joint gap can induce repair in the TMJ region, similar to that in unstable fracture healing. The process by which Hippo–YAP signalling induces the osteogenic and chondrogenic lineage differentiation of MSCs is regulated by different forces applied from the environment^36^, especially shear stress^37,38^. Zhong et al.^37^ found that shear stresses enhance YAP expression, ultimately resulting in an increase in osteogenesis, a decrease in adipogenesis in MSCs and the initiation of dedifferentiation in chondrocytes. Therefore, we hypothesise that MSCs can convert mechanical stimuli into biochemical signals and that signalling molecules such as YAP promote the osteogenic differentiation of MSCs in response to shear stress.

In response to cyclic shear stress, the formation of an ankylosed callus is dominated by endochondral ossification^5^, producing a structure similar to that in hypertrophic nonunion^4^, which is rich in fibrocytes and nonmineralised chondrocytes^39,40^. Interestingly, YAP promoted the expression of SOX6, which is required for chondrocyte proliferation, but suppressed the expression of COL10A (a hypertrophic chondrocyte marker)^23^. Similarly, Yang et al.^41^ reported that YAP enhances chondrocyte proliferation but inhibits chondrocyte hypertrophic differentiation. These data indicate that YAP has different effects at each stage during the process of mediating endochondral ossification in bone repair: promoting chondrocyte proliferation and differentiation at the early stage and hindering the final hypertrophic differentiation at the later stage. We surmise that after TMJ trauma, the effect of YAP on chondrocytes in the joint region is also in accordance with the above mechanism of producing structures similar to those involved in hypertrophic nonunion.

The proliferation ability of stem cells is the basis of all regenerative activities^42,43^, and the migration ability of stem cells is indispensable for tissue regeneration^44^. The results of this study showed that silencing Hippo–YAP signalling inhibits MSC proliferation, and an additional by Tang^45^ demonstrated this phenomenon. In the Tang study, after YAP was knocked down in human peripheral ligament stem cells, cell proliferation significantly decreased and the apoptosis rate increased. Knocking down YAP also inhibited migration, which is consistent with the role of the Hippo–YAP signalling pathway in tumorigenesis^46^ and with the function of the pathway itself^47^.

Research on the silencing of Hippo–YAP was limited to in vitro cell culture in this study, and its role in the process of TMJ posttraumatic repair is still unclear. Gong et al.^48^ used an intra-articular injection of YAP-interfering RNA to alleviate osteoarthritis (OA). In vivo injection of small interfering RNAs could compensate for the shortcomings of our study. In the future, the silencing sequence can be screened and verified via in vivo experiments, and the confirmed RNA sequence can be used as a drug for intra-articular injection to observe its effect on traumatic bony ankylosis of the TMJ.

This study has several limitations. First, small sample sizes (n = 3) produced underpowered analyses for some TMJ ankylosis outcome measures in the sheep model (the mIHC staining, in particular), which may cause a significant number of type 2 errors in the statistical analysis. Second, we showed that YAP can affect the function of MSCs, especially osteogenic differentiation; however, the mechanism underlying these effects is not yet clear and requires further research.

## Conclusions

We demonstrated that the Hippo–YAP pathway is involved in every early stage of the pathological process of traumatic TMJ ankylosis. Hippo–YAP signalling is involved in the osteogenic process of MSCs. The inhibition of YAP via YAP shRNA can inhibit the osteogenic, proliferation and migration potential of MSCs and ultimately reduce bone formation. This study provides a theoretical basis for exploring the molecular mechanisms underlying TMJ ankylosis. Our findings indicate that the inhibition of YAP using YAP shRNA can be an effective way to prevent TMJ ankylosis.

## Data Availability

The datasets used and/or analysed during the current study are available from the corresponding author on reasonable request.
